# Identification of Discrete Sites in Yip1A Necessary for Regulation of Endoplasmic Reticulum Structure

**DOI:** 10.1371/journal.pone.0054413

**Published:** 2013-01-14

**Authors:** Kaitlyn M. Dykstra, Idil Ulengin, Nicholas DelRose, Tina H. Lee

**Affiliations:** Department of Biological Sciences, Carnegie Mellon University, Pittsburgh, Pennsylvania, United States of America; Ecole Polytechnique Federale de Lausanne, Switzerland

## Abstract

The endoplasmic reticulum (ER) of specialized cells can undergo dramatic changes in structural organization, including formation of concentric whorls. We previously reported that depletion of Yip1A, an integral membrane protein conserved between yeast and mammals, caused ER whorl formation reminiscent of that seen in specialized cells. Yip1A and its yeast homologue Yip1p cycle between the ER and early Golgi, have been implicated in a number of distinct trafficking steps, and interact with a conserved set of binding partners including Yif1p/Yif1A and the Ypt1/Ypt31 Rab GTPases. Here, we carried out a mutational analysis of Yip1A to obtain insight into how it regulates ER whorl formation. Most of the Yip1A cytoplasmic domain was dispensable, whereas the transmembrane (TM) domain, especially residues within predicted TM helices 3 and 4, were sensitive to mutagenesis. Comprehensive analysis revealed two discrete functionally required determinants. One was E95 and flanking residues L92 and L96 within the cytoplasmic domain; the other was K146 and nearby residue V152 within the TM domain. Notably, the identified determinants correspond closely to two sites previously found to be essential for yeast viability (E76 and K130 in Yip1p corresponding to E95 and K146 in Yip1A, respectively). In contrast, a third site (E89) also essential for yeast viability (E70 in Yip1p) was dispensable for regulation of whorl formation. Earlier work showed that E76 (E95) was dispensable for binding Yif1p or Ypt1p/Ypt31p, whereas E70 (E89) was required. Collectively, these findings suggest that the ability of Yip1A to bind its established binding partners may be uncoupled from its ability to control ER whorl formation. In support, Yif1A knockdown did not cause ER whorl formation. Thus Yip1A may use the sites identified herein to interact with a novel binding partner to regulate ER membrane organization.

## Introduction

The ER is a singular and essential organelle with a complex three-dimensional structure. It consists of both flattened sheet-like cisternal membranes and highly curved tubules that are interconnected at hundreds of three-way junctions [1]. In most cell types, ER membranes are widely distributed throughout the cell cytoplasm, extending from the outer nuclear envelope to the cell periphery [2]–[4].

Many essential processes, including protein and lipid biosynthesis, drug detoxification and calcium regulation, occur within sub-domains of the ER [3]. In response to specific developmental cues, select sub-domains of the ER undergo dramatic expansion, presumably reflecting physiological changes in demand for certain ER functions over others [5]. The ER can also undergo major changes in overall organization. For instance, in professional secretory pancreatic acinar cells, flattened sheets of ribosome-studded rough ER membranes are organized into regular parallel arrays [3], [6]. In other specialized cell types that secrete either peptide or steroid hormones, rough or smooth ER membranes undergo reversible reorganization into concentric ribbon-like whorls [7]–[9]. In many cases, neither the mechanisms that alter ER organization, nor the functional consequences on organelle function, are well understood.

We previously identified the ER-to-Golgi cycling protein Yip1A as a regulator of ER network structure and organization. RNAi mediated knockdown of Yip1A in HeLa cells resulted in a remarkable transformation of the typically dispersed ER network into tightly stacked, micrometer sized concentric membrane whorls [10]. Importantly, the ER whorl phenotype, somewhat reminiscent of the ribbon-like concentric whorls seen in specialized cells [7]–[9], was specific to the loss of Yip1A, as it was rescued by the expression of a siRNA immune Yip1A construct [10].

Our identification of Yip1A as an apparent ER structuring protein was surprising in several respects. First, although as much as half the protein is present in the ER at any given time [11], Yip1A undergoes constant ER exit and depends on retrieval from post-ER compartments to achieve its steady state ER exit site localization [12], [13]. Second, Yip1A was initially discovered as a yeast protein required for vesicle trafficking rather than organelle structuring. In one set of studies yeast Yip1p was implicated in COPII-mediated vesicle biogenesis [12]; while in another, Yip1p was implicated in fusion of ER-derived COPII vesicles with the Golgi [14]. Consistent with its ER-to-Golgi cycling behavior, mammalian Yip1A was shown to bind the Sec23/24 subunit of the COPII coat [15]; and furthermore, stable binding partners of Yip1p have been identified in Yif1p [16] and Yos1p [11], also ER-to-Golgi cycling proteins. Additional though likely more transient interacting partners have been found in Yop1p [17] and the Ypt1p/Ypt31p sub-class of Rab GTPases [18], [19]. Finally, mammalian Yip1A was also found to be required for COPI-independent retrograde trafficking to the ER [20]. Consistent with earlier work implicating Yip1p/Yip1A in trafficking between the ER and Golgi, our work also revealed a marked delay of COPII-mediated protein export from the ER in HeLa cells depleted of Yip1A [10]. However, the delay could in principle be attributed to a secondary consequence of ER whorl formation, as whorl formation through an entirely independent means [21] was sufficient to delay ER export [10].

Whether Yip1A plays a direct or indirect role in regulating ER whorl formation remains to be determined. In addition to naturally occurring instances of whorl formation in specialized tissues [7]–[9], ER whorl formation has been observed upon experimental up-regulation of a variety of membrane-anchored proteins such as cytochrome P450 [22], HMG-CoA reductase [23], microsomal aldehyde dehydrogenase [24], cytochrome b5 [25], the inositol 1,4,5-triphosphate receptor [26] and the simultaneous over-expression of VapB and its binding partner Nir2 [27]. In each instance, membrane stacking and whorl formation seem to be driven largely by self-association of the cytoplasmic domain of the over-expressed ER membrane-anchored protein in trans [21]. Whether a similar mechanism underlies ER whorl formation after Yip1A loss remains unknown. And if so, the identity of the protein(s) that might undergo trans interactions to bring about ER stacking and whorl formation also remains to be determined.

In this study, we wished to gain insight into the molecular mechanism by which Yip1A regulates ER structure. Taking advantage of the ability of a siRNA-immune Yip1A transgene to rescue the whorled ER phenotype in knockdown cells [10], we carried out a systematic mutational analysis of nearly all residues in the protein; our goal being to determine those residues most important for its ER structural maintenance role.

## Materials and Methods

### Cell culture and transfections

HeLa cells stably expressing a GalNacT2-green fluorescent protein (GFP) [28] were maintained in minimal essential medium (Sigma-Aldrich, St. Louis, MO) containing 10% fetal bovine serum (Atlanta Biologicals, Norcross, GA) and 100 IU/ml penicillin and streptomycin (Mediatech, Herndon, VA) at 37°C in a 5% CO_2_ incubator. Transient plasmid DNA transfection of HeLa cells was performed with jetPEI™ (Polyplus transfection, Illkirch, France), according to the manufacturer's specifications using 0.5 μg DNA per 1 mL media. Transient co-transfection of HeLa cells with both plasmid DNA and siRNA was performed with jetPRIME™ (Polyplus transfection) according to the manufacturer's specifications by using 150 ng of DNA and 10 pmol siRNA per 0.5 mL media. Transient siRNA transfections of siRNAs against Yif1A were performed using jetPRIME™ (Polyplus transfection) using 20 pmol siRNA per 0.5 mL media.

### Quantification of efficiency of rescue

For each of our transgene replacement experiments, at least 100 cells were counted in three individual experiments and the data was calculated as the percentage of cells expressing the transgene that display ER whorls. In order to compare the different constructs over multiple sets of experiments, this percentage was normalized to the negative control for that experiment using the following formula: Efficiency  =  1 – (fraction cells expressing transgene with ER whorls/fraction cells expressing negative control with ER whorls). An efficiency of 1 reflects full rescue and 0 is a complete non-rescue.

### Constructs

The siRNAs used against Yip1A were described previously (10) and synthesized by Ambion (Austin, TX). The myc-tagged Sec61β was subcloned from GFP-Sec61β (kindly provided by Dr. Tom Rapoport, Harvard Medical School, Boston, MA) into the EcoRI and XbaI sites of the pCS2-MT vector. The myc-tagged Yif1A construct was cloned by PCR amplification from HeLa cDNA (Qiagen, Hilden, Germany) and inserting the PCR product into the pCS2-MT vector using EcoRI and XbaI sites. The HA-Yip1A rescue construct was created by replacing the FLAG epitope from the FLAG-Yip1A construct [10] with the HA epitope (YPYDVPDYA) using a PCR-based loop-out/loop-in modification of the QuikChange protocol (Stratagene, La Jolla, CA) and was the parent construct for all further HA-Yip1A mutations. The HA-Yip1AN/Sec61β TM was created by first using a PCR-based loop-out technique (Stratagene) to remove the TM domain region (AA 126–257) of the Yip1A construct and the TM domain from Myc- Sec61β (AA 61–97) was subcloned into the XbaI site at the C-terminus. The HA-Yip1A Δ1-83 and Δ1-118 constructs were created using the PCR-based loop-out technique (Stratagene). All additional HA-Yip1A mutant constructs were created using QuikChange site directed mutagenesis PCR (Stratagene). siRNAs directed against Yif1A were created using a siRNA construction kit (Ambion) and previously published target sequences [13]. The control siRNA used in this study targets bovine p115 and does not affect p115 in HeLa cells [29].

### Antibodies, immunofluorescence and immunoblotting

Antibodies used include mouse monoclonal antibody (mAb) against the HA-epitope (Sigma-Aldrich, St. Louis, MO); a rabbit polyclonal antibody (pAb) against Calnexin, a pAb against tubulin and a mAb against protein disulfide isomerase (PDI) (both Abcam, Cambridge, MA); the 9E10 mAb against the myc epitope [30]; a pAb against GPP130 (kindly provided by Dr. A. Linstedt, Carnegie Mellon University, Pittsburgh, PA). Fluorophore-conjugated secondary antibodies were from Zymed Laboratories (South San Francisco, CA)/Invitrogen (Carlsbad, CA). HeLa cells were typically analyzed 72 h post-transfection. Immunofluorescence procedures were as described previously [10]. Immunoblotting using a mouse mAb against the myc-epitope and a rabbit pAb against tubulin (Abcam), was performed on cells co-transfected with Yif1A siRNA and myc-Yif1A harvested from 60-mm dishes as described previously [31].

### Fluorescence microscopy

All images were obtained using a Yokagawa spinning disk confocal scanhead (Perkin Elmer Life and Analytical Sciences, Boston MA) mounted on an Axiovert 200 microscope (Carl Zeiss, Jena, Germany) with a 100X 1.4 numerical aperture (NA) objective (Carl Zeiss) and acquired using a 12-bit Orca ER digital camera (Hamamatsu Photonics, Hamamatsu City, Japan). Maximal value projections of sections at 0.3-μm spacing (5–8/cell) were acquired using ImageJ (National Institutes of Health, Bethesda, MD).

## Results

### Both the cytoplasmic and transmembrane domains of Yip1A are required for ER structural maintenance

**Figure 1:**
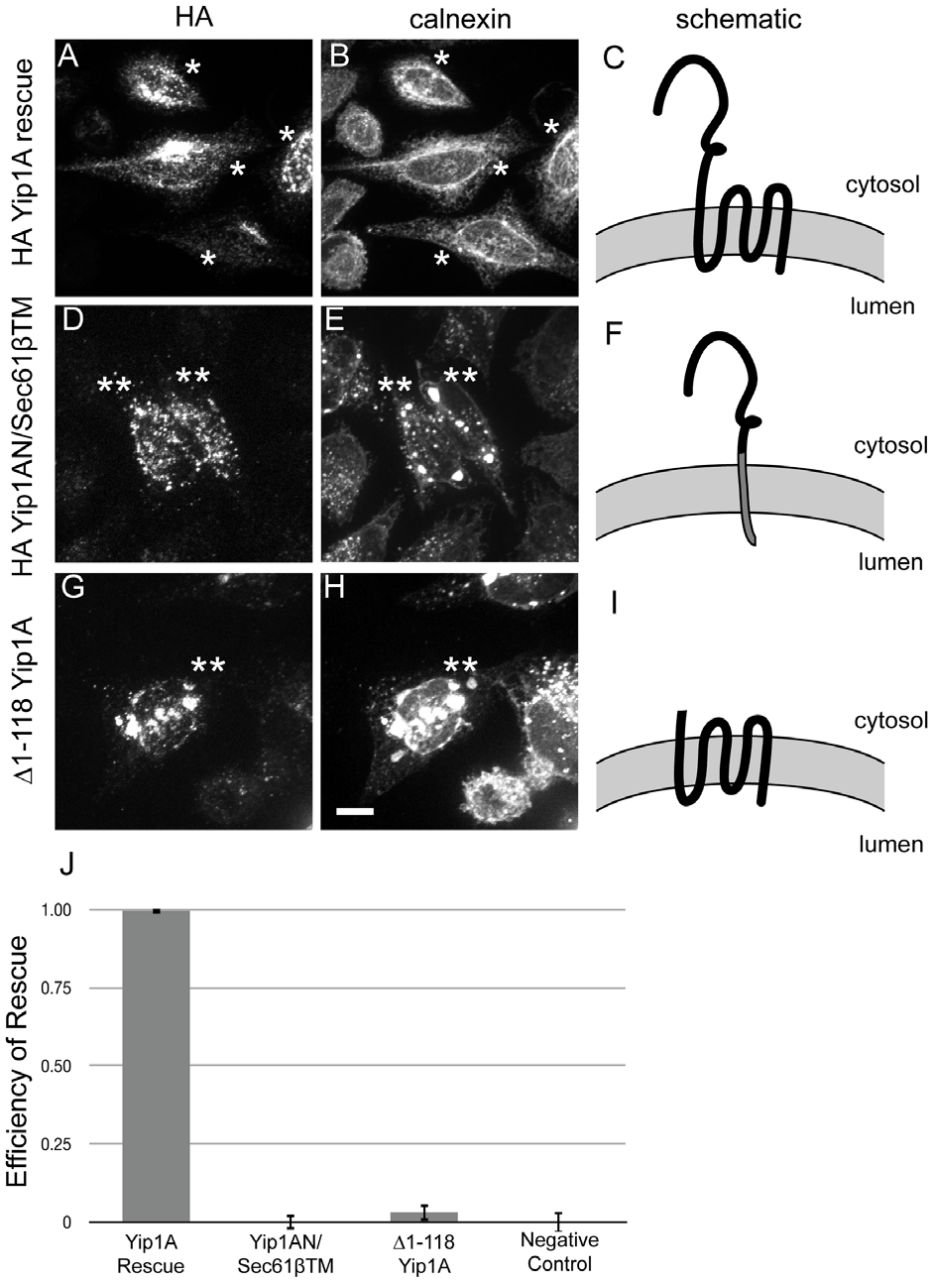
Both the cytoplasmic and TM domains of Yip1A are required to regulate ER whorl formation. HeLa cells were co-transfected with Yip1A siRNA and either a negative control myc-Sec61β construct (not shown), a wild-type HA-Yip1A rescue construct (A–C), a chimeric construct (HA-Yip1AN/Sec61βTM) with the N-terminus of Yip1A fused to the TM helix of Sec61β (D–F), or a Yip1A truncation construct (HA-Yip1A Δ1–118) lacking the entire cytoplasmic domain (G–I). Cells were fixed 72 h after transfection and co-stained with antibodies against HA (A, D, G) or Myc (not shown) and calnexin (B, E, H). Single asterisks mark cells expressing the indicated construct that did not exhibit ER whorls; whereas double asterisks mark expressing cells that did exhibit ER whorls. Scale bar, 10 μm. The constructs are schematized (C, F, I). (J) The normalized efficiency of rescue by each variant was quantified as detailed in Materials and Methods. Data were from 3 independent experiments (>100 cells per experiment), ±SD.

We began by testing the cytoplasmic and transmembrane (TM) domains of Yip1A independently for their ability to regulate ER whorls. Two constructs based on the siRNA-immune HA-Yip1A parent rescue construct were generated (schematized in Fig. 1C). One replaced the entire C-terminal multi-pass TM domain of Yip1A with the single pass C-terminally anchored TM domain of Sec61β [32] so as to maintain ER membrane targeting of the cytoplasmic domain (HA-Yip1AN/Sec61βTM; schematized in Fig. 1F). The other truncated the entire N-terminal cytoplasmic domain (HA-Yip1A Δ1–118; schematized in Fig. 1I). Each construct was transiently transfected into HeLa cells together with the Yip1A siRNA and processed 72 hrs later for immunofluorescence using antibodies against the HA epitope and the integral ER membrane marker calnexin [33]. As expected, HA-Yip1A displayed primarily an ER exit site pattern at steady state (Fig. 1A) with some detectable overlap with the ER marker calnexin (Fig. 1B). Similar to our previous report [10], few if any cells expressing wild type HA-Yip1A displayed whorls (Fig. 1J) while 68±3% of cells expressing the negative control construct Myc-Sec61β had ER whorls (Fig. 1J). This was as expected for full rescue by the wild type HA-Yip1A transgene. For ease of comparison across experiments, we present the data from hereon in terms of rescue efficiency (calculated as detailed in Materials and Methods), with 1 representing full rescue as exhibited by wild type Yip1A and 0 representing non-rescue as exhibited by the negative control Myc-Sec61β. Quantification in this manner revealed that neither HA-Yip1AN/Sec61βTM (Fig. 1D, E; quantified in J) nor HA-Yip1A Δ1-118 (Fig. 1G, H; quantified in J) could rescue the ER whorl phenotype; indeed both were indistinguishable from the negative control. Thus Yip1A depends on both its cytoplasmic and TM domains for function.

Of note, HA-Yip1AN/Sec61βTM, lacking the entire Yip1A TM domain, seemed to exhibit less overlap with the ER marker calnexin than did full-length HA-Yip1A (compare Fig. 1D, E to Fig. 1A, B). Conversely, HA-Yip1A lacking its entire cytoplasmic domain seemed to have greater overlap with calnexin (compare Fig. 1G, H to Fig. 1A, B). These differences likely reflected a shift in the steady state distribution of each deletion variant with respect to full-length HA-Yip1A. That is, deletion of the Yip1A TM domain appeared to dispose the chimeric protein more towards post-ER compartments; while deletion of the cytoplasmic domain appeared to dispose the truncated protein more towards the ER. This raised a caveat that the inability of HA-Yip1AN/Sec61βTM to control ER whorl formation might not be due to loss of a determinant required for regulating whorl formation, per se; but rather, to its sequestration from whorl forming membranes. Importantly though, subsequent detailed mapping of functional determinants within the TM domain indicated that Yip1A does indeed depend on residues within its TM domain for regulating whorl formation (see below). Thus the apparent lack of ER structuring activity by HA-Yip1AN/Sec61βTM likely reflects a required role for the Yip1A TM domain in regulating ER whorl formation.

### Only a few key residues comprising a single site in the cytoplasmic domain are required

**Figure 2:**
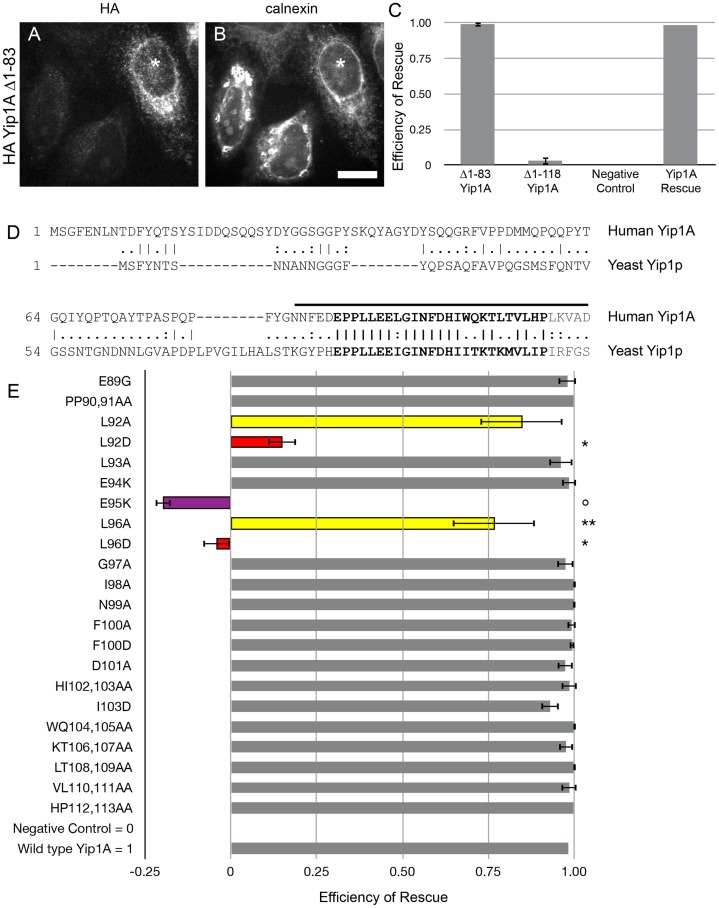
Only a few highly conserved residues in the Yip1A cytoplasmic domain are required for function. Cells co-transfected with Yip1A siRNA and HA-Yip1A Δ1-83 were fixed after 72 h and co-stained with antibodies against HA (A) and calnexin (B). The asterisk (A and B) marks an expressing cell that did not exhibit ER whorls. Scale bar, 10 μm. (C) Quantification of the efficiency of rescue by HA-Yip1A Δ1–83, HA-Yip1A Δ1–118 and the negative control Myc-Sec61β. Data were from 3 independent experiments (>100 cells per experiment), ±SD. (D) An alignment of the cytoplasmic domains of human Yip1A with yeast Yip1p. Residues 83–118 are bracketed, with the highly conserved block highlighted in bold. (E) Bolded residues (in D) were mutated as indicated and tested for rescue. Data from 3 independent experiments (>100 cells per experiment) ±SD are quantified. Yellow bars indicate residues that were partially functional when mutated to alanine. Red bars indicate those same residues showing a more significant loss of function when mutated to a charged residue. Single asterisk, p-value <0.001; double asterisk, p<0.05 (Student's t-test). The open circle and purple bar indicate the previously identified nonfunctional variant E95K [10].

Given that the cytoplasmic and TM domains of Yip1A both appeared to be required for function, we sought to define the necessary elements in each half, starting with the cytoplasmic domain. We previously showed that a conserved Glu residue (E95) required for Yip1p-dependent viability in yeast [19] was also required for the ER structuring function of Yip1A in HeLa cells [10]. However, we wanted to extend our prior analysis by determining if the Yip1A cytoplasmic domain contained any other residues essential for function. We began with progressive truncations from the N-terminus of the protein, testing each truncated version for function. Deletion of the first 83 amino acids had no observable effect on function, with virtually all cells expressing the truncated protein having a normal dispersed ER morphology (Fig. 2A, B and quantified in C). This suggested that the first 83 amino acids are dispensable for the ER structuring function of Yip1A. This was consistent with an earlier observation that the first 65 residues of the yeast homolog Yip1p are dispensable for yeast viability [19]. However, as indicated above, further truncation to residue 118 (the end of the cytoplasmic domain) resulted in a non-functional, though stably expressed protein (Fig. 1G, H; quantified in J), narrowing the required elements in the cytoplasmic domain to residues lying between 83 and 118.

An alignment between the yeast and human Yip1 sequences revealed a highly conserved block of residues between the human residues 89 and 113 (Fig. 2D). We proceeded to replace either individual or pairs of residues in the conserved block with Ala (unless otherwise indicated in Fig. 2E) and tested for function. The results of this analysis are quantified in Fig. 2E. Significantly, the E89G mutation – corresponding to the lethal *yip1-41* allele in yeast (E70G in Yip1p) previously shown to abolish binding of Yip1p to Yif1p as well as to Ypt1p and Ypt31p [19] – was fully functional for ER structural maintenance by Yip1A. This was surprising and suggested that the control of ER whorl formation by Yip1A might not require binding to either Yif1A or the Rab GTPases. On the other hand, the previously identified Glu residue mutated to Lys (E95K) proved to be most disruptive to function; E95K was the only mutant variant that had a significant dominant negative effect in our rescue assay (Fig. 2E). This mutation corresponds to another lethal allele of Yip1p, *yip1-6* (E76K in Yip1p); though in contrast to *yip1-41*, it had only minor effects on binding of Yip1p to either Yif1p or Ypt1p/Ypt31p [19]. Collectively, these data reveal that the ability of Yip1A to regulate ER structure does not correlate with its ability to bind either Yif1A or Rab GTPases.

Interestingly, we saw a mild loss of function when two Leu residues (L92 and L96) surrounding the essential E95 residue were replaced with Ala. Because a Leu to Ala substitution is a relatively conservative change, we further tested the effect of replacing L92 or L96 with a charged residue. Indeed, both L92D and L96D yielded essentially nonfunctional proteins, indicating the importance of the uncharged character of those residues for function (Fig. 2E). On the other hand, a similar substitution of other nearby conserved nonpolar residues such as F100D or I103D had no effect (Fig. 2E). This selectivity underscores the importance of L92 and L96 in addition to E95. As L92, E95 and L96 are predicted to lie on the same face of a predicted short alpha helix, it is tempting to speculate that the three residues comprise a single binding determinant on Yip1A.

### Ala/Leu replacements of transmembrane segments reveal crucial residues

**Figure 3:**
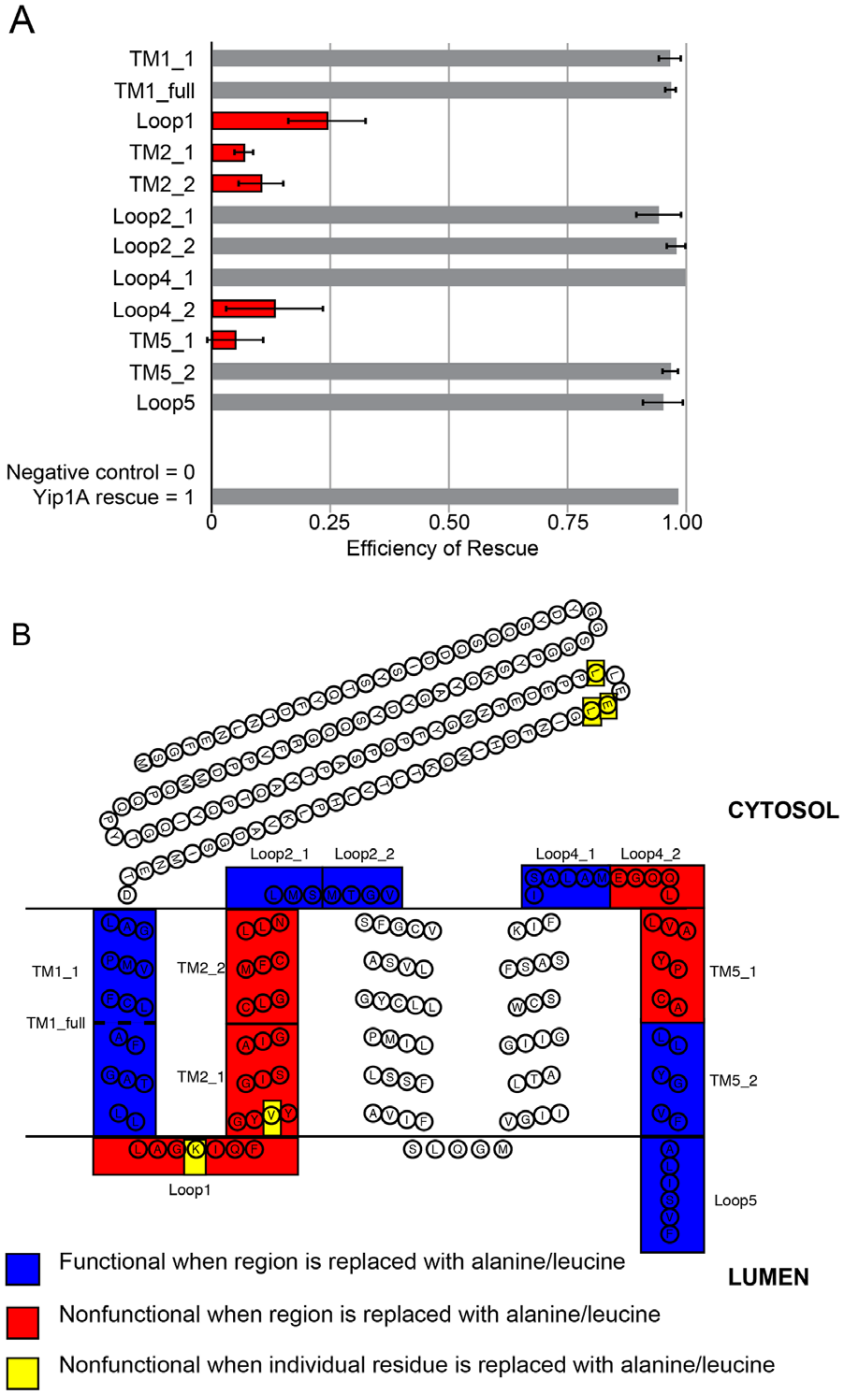
Regions of the Yip1A TM domain required for ER structuring. (A) Quantification of rescue in cells that were co-transfected with Yip1A siRNA and mutated HA-Yip1A constructs. Data were from 3 independent experiments (>100 cells per experiment), ±SD. Red bars indicate regions that were nonfunctional when mutated to Ala/Leu. (B) A schematic representation of the predicted topology of Yip1A. The results from (A) are represented on the schematic. Residues highlighted in blue were functional and red were nonfunctional when stretches of amino acids were replaced with Ala/Leu. Residues highlighted in yellow were nonfunctional when individual amino acids were replaced with Ala/Leu. Precise substitutions are detailed in Table S1.

For dissecting the required regions of the TM domain, we relied on both secondary structure and TM domain prediction algorithms (schematized in Fig. 3B) to make truncations of individual TM helices and loops so as to minimize perturbation of overall membrane topology. Unfortunately, none of the resulting deletion constructs led to stably expressed protein (data not shown). To bypass this issue, we proceeded by replacing segments of predicted TM helices with Ala residues interspersed with Leu so as to maintain their stability in the membrane [34]; similarly, predicted luminal and cytoplasmic loops were replaced with stretches of Ala. In the case of charged residues, charge reversal mutations were made to maximize the potential effect of the mutation. All of the constructs made were expressed, except for those with substitutions in TM3 and TM4. Curiously, even single amino acid substitutions within TM3 and TM4 led to poorly expressed protein, precluding their analysis. Nonetheless all other TM helices and loops were amenable to substitutions, allowing a dissection of the remainder of the TM domain.

**Figure 4:**
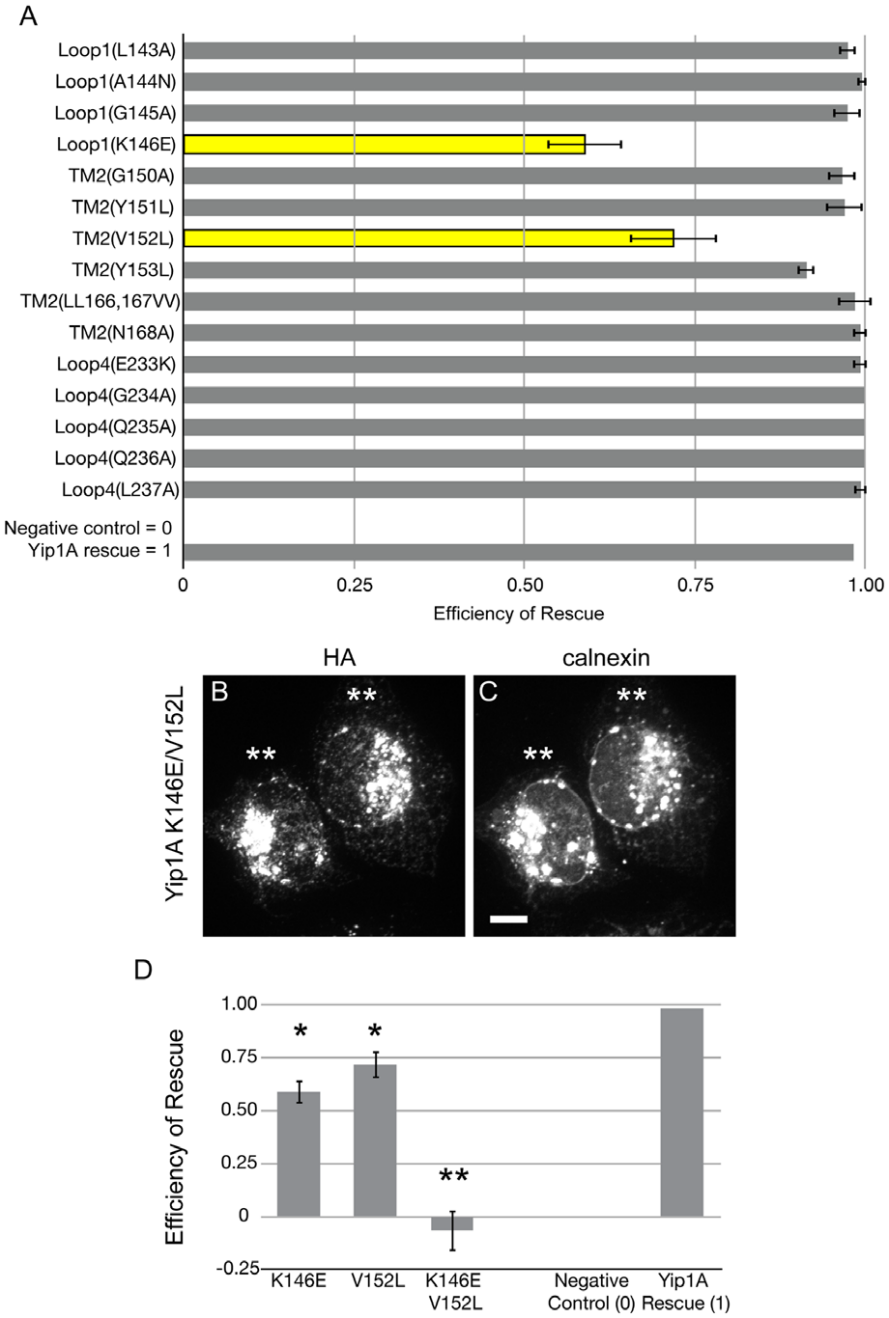
Two residues within the Yip1A TM domain are essential for the ER structuring function of Yip1A. (A) Quantification of cells that were co-transfected with the indicated HA-Yip1A mutated constructs and Yip1A siRNA. Data were from 3 independent experiments (>100 cells per experiment), ±SD. Yellow bars indicate mutations that resulted in a partial rescue. (B, C) Cells co-transfected with Yip1A siRNA and HA-Yip1A K146E and V152L single or double mutant variant constructs were fixed after 72 h and co-stained with HA (B) and calnexin (C) antibodies. Double asterisks indicate cells expressing the double mutant variant that exhibited ER whorls. Scale bar, 10 μm. (D) Quantification of the efficiency of rescue for (B) and (C) from three independent experiments (>100 cells per experiment) ±SD. Single asterisk, p≤0.02 and double asterisk, p<0.0001.

Each expressing construct was tested in our knockdown replacement assay and the results are quantified in Fig. 3A and schematized in Fig. 3B. (A full table of mutations and results is included in Table S1). The analysis revealed substantial stretches where residue identity was seemingly not important for function, such as all of TM1 and Loop2; however for others, such as TM2 and Loop1, substitutions of 7–10 amino acid stretches disrupted function. To further refine the required residues, we constructed point mutations within the seemingly required regions. Surprisingly, when variants with individual amino acid substitutions were produced, it became clear that only two point mutations, K146E and V152L, resulted in any loss of function, though the loss was modest (Fig. 4A, D). Given their proximity to one another, we also tested the effect of combining the two substitutions. Notably, the double mutant variant K146E/V152L was completely nonfunctional (Fig. 4B, C and quantified in D), suggesting that the two residues might work in a cooperative fashion to support Yip1A function. This was intriguing given that the yeast mutant counterpart of K146E (K130E) was similarly disruptive to Yip1p function when combined with another mutation [19]. Importantly, we confirmed that the loss of function of these and other key non-rescuing variants described above was not due to low protein expression levels (Fig. S1). In sum, though multiple large-scale substitutions within the TM domain of Yip1A were disruptive, the identity of only a few individual residues, namely K146 in the first luminal loop, and V152 – a nearby residue at the start of the second TM helix – were clearly necessary for regulation of ER whorl formation.

### Required residues in Yip1A may control ER whorl formation independently of its established binding partners Yif1A/Yif1p and the Ypt1p/Ypt31p subclass of GTPases

**Figure 5:**
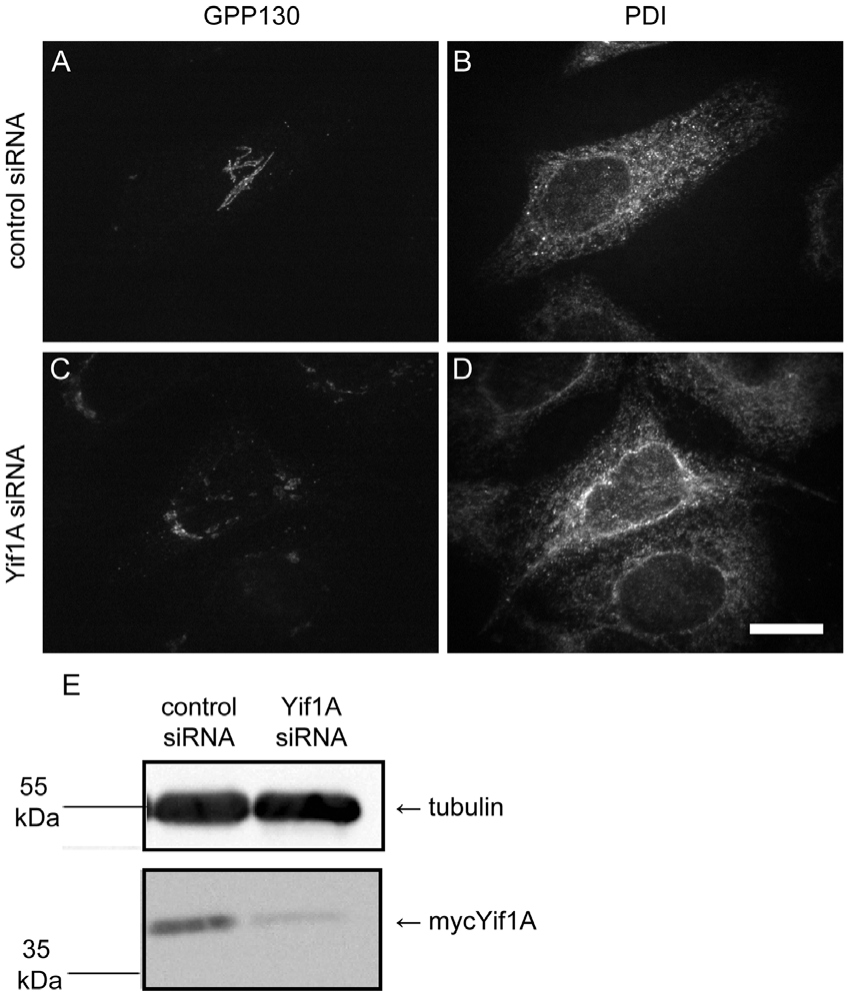
Yif1A knockdown does not result in a whorled ER phenotype. HeLa cells transfected with either a negative control siRNA (A and B) or siRNA against Yif1A (C and D) were fixed after 72 h and costained with antibodies against GPP130 (A and C) and PDI (B and D). (E) HeLa cells co-transfected with mycYif1A and either a control siRNA or Yif1A siRNA, were harvested after 72 h and then immunoblotted using antibodies against tubulin and the myc-epitope.

It was initially surprising that our unbiased analysis revealed in essence only two discrete sites crucial for the ER structural maintenance function of Yip1A (residues centered around E95 and K146). However it was also satisfying that the identified sites corresponded precisely to two sites previously shown to be essential for Yip1p-dependent viability in yeast (E76 and K130). More surprising was our finding that a third site previously shown to be essential for Yip1p function in yeast (E70) was completely dispensable for the control of ER whorl formation by Yip1A. This suggested that Yip1A/Yip1p might possess two separate functions. One function – supported by E95 (E76 in yeast) and its flanking residues L92 and L96, as well as K146 (K130 in yeast) and nearby residue V152 – that is required for ER structural maintenance; and another, supported by E89 (E70), that is dispensable for ER structural maintenance. Furthermore, the ability of Yip1p to bind its established binding partners Yif1p and Ypt1p/Ypt31p, mapped to E70 [19], the residue dispensable for control for ER whorl formation by Yip1A. Thus it seemed that the ER structuring function of Yip1A/Yip1p might operate independently of either Yif1A/Yif1p or the Ypt1p/Ypt31p category of Rab GTPases. Pertaining to this hypothesis, RNAi-mediated Yif1A knockdown was previously reported to cause a fragmentation of the Golgi apparatus, consistent with an ER-to-Golgi trafficking defect, but no ER phenotype was reported [13]. To support our conjecture that Yip1A-mediated control of ER whorl formation does not depend on Yif1A, we reproduced the previously published Yif1A knockdown but with an eye towards revealing any potential ER phenotypes. Consistent with a lack of requirement for Yif1A in ER structural maintenance, we observed no ER whorls in cells depleted of Yif1A, even though Golgi fragmentation was clearly and frequently observed (Fig. 5).

## Discussion

### The residue identity of surprisingly few amino acids tested are important for the mechanism of Yip1A function

As shown previously for its yeast counterpart in a viability assay [19], the majority of the cytoplasmic domain of Yip1A was dispensable for its ER structural maintenance function. This dispensable portion has no predicted secondary structure; therefore it is difficult to speculate on its potential role. Yet its persistence through evolution suggests a role, perhaps regulatory, under conditions yet-to-be assessed. In contrast to the cytoplasmic domain, we found that the membrane-spanning domain of Yip1A was extremely sensitive to deletion mutagenesis. This was especially the case for TM3 and TM4, where even single amino acid substitutions severely compromised protein stability. We speculate that the five predicted TM helices pack together to adopt a stable tertiary structure, with TM3/4 at the core. As we were unable to generate any stably expressing variants within TM3/4, it is unclear whether individual residues within this region are required specifically for the ER structural maintenance function of Yip1A, or whether they might simply play a scaffolding role for protein folding and stabilization.

Unlike TM3 and TM4, the remaining predicted helices TM1, TM2 and TM5 could be extensively mutagenized without compromising protein stability. Indeed, the entirety of TM1 and the latter half of TM5 could be replaced by Ala/Leu residues, indicating that TM1 and the second half of TM5 are unimportant either for protein stability or for protein function. In contrast, TM2 and the first half of TM5 seemed functionally important at first. Their substitution with stretches of Ala/Leu yielded nonfunctional though stably expressed protein. However, point mutations within these regions failed to identify individual residues crucial for function. We can envision two straightforward explanations for this apparent discrepancy. First, the entire length of TM2 may pack in a specific way against TM3/4/5, via a relatively large binding interface, to adopt a tertiary structure required for function. And therefore, while large-scale substitutions in TM2 (or TM5) might be deleterious to protein function because they would compromise the helix packing, individual point mutations may not be sufficiently disruptive to helix packing to undermine protein stability and function. A second possibility, not incompatible with the first, is that Ala/Leu replacement is a relatively conservative change for membrane-spanning residues. Hence, additional required residues may have been missed in our analysis.

A comprehensive scan of the remainder of the Yip1A membrane spanning domain as well as its cytoplasmic domain revealed only a surprisingly few amino acids whose identity was crucial for function: residues predicted to lie on one face of a predicted short alpha helix in the cytoplasmic domain (L92, E95, L96) and those within the first luminal loop and adjacent TM2 helix (K146 and V152). As Yip1A lacks any identifiable structural motifs indicative of function, we speculate that these residues interface either with a required protein-binding partner and/or directly with the phospholipid bilayer to regulate ER whorl formation.

### Control of ER structure by Yip1A is likely independent of its established binding partners

It is revealing that a mutation (E89 in human and E70 in yeast) that abolishes Yip1p binding to either Yif1p or Ypt1p/Ypt31p GTPases [19] had no impact on the ability of Yip1A to regulate ER whorl formation; whereas mutations (E95 and K146 in human and E76 and K130 in yeast) that have minor if any effects on Yip1p binding to either Yif1p or Ypt1p/Ypt31p [19] were completely disruptive. As both sets of mutations are lethal for yeast, it seems reasonable to speculate that Yip1p/Yip1A has at least two distinct essential functions: one that depends on Yif1p and Ypt1p/Ypt31p binding; and a separate function in regulating ER structure that does not depend on the same binding partners.

### How might Yip1A control ER whorl formation?

Candidate Yip1A/Yip1p binding partners additional to Yif1A/Yif1p and Ypt1p/Ypt31p GTPases [16], [18] include the curvature-inducing integral ER membrane protein Yop1p/DP1 [17], [35]. We previously reported that the nonfunctional E95K mutant variant of Yip1A retains binding to DP1 [10], the mammalian homologue of Yop1p [35]. This was also the case for the K146E/V152L mutant variant (data not shown). Thus, none of the previously identified Yip1A/Yip1p binding partners are obvious candidates for mediating the ER structural maintenance role of Yip1A. A final intriguing possibility is that Yip1A affects ER membrane morphology through a direct lipid interaction. As little is understood about how local lipid composition contributes to the structure of the ER, it seems plausible that Yip1A might directly bind and sort lipids thereby maintaining an ER membrane composition that is conducive to a dispersed, rather than stacked, membrane network. Alternatively, Yip1A could direct localized lipid synthesis by binding and regulating a lipid-modifying enzyme. Intriguingly, Got1p, a high copy suppressor of a temperature sensitive Yip1p mutant in yeast has been proposed to affect lipid composition [36]. These possibilities have yet to be explored, and the identification of two crucial functional determinants in this study will be useful for future mechanistic studies of the control of ER whorl formation by Yip1A.

## Supporting Information

Figure S1 **Nonfunctional mutant variants of HA-Yip1A are expressed at levels similar to wild type HA-Yip1A.** HeLa cells transfected with the indicated HA-Yip1A variants were fixed 48 h later, stained with antibodies against the HA epitope, and the total fluorescence intensity per cell measured in ImageJ. The data for 50–100 random cells were binned according to levels of fluorescence and plotted in a histogram as the percent of cells exhibiting the indicated levels of fluorescence.
(TIF)

Table S1 **All Yip1A variants assessed in this study.** For each mutant variant, the precise amino acid change, subcellular localization and efficiency of rescue are indicated.
(XLS)

## References

[1] Voeltz GK, Rolls MM, Rapoport TA (2002) Structural organization of the endoplasmic reticulum. EMBO Rep 3: 944–950. 12370207 10.1093/embo-reports/kvf202 PMC1307613

[2] Terasaki M, Chen LB, Fujiwara K (1986) Microtubules and the endoplasmic reticulum are highly interdependent structures. J Cell Biol 103: 1557–1568. 3533956 10.1083/jcb.103.4.1557 PMC2114338

[3] Baumann O, Walz B (2001) Endoplasmic reticulum of animal cells and its organization into structural and functional domains. Int Rev Cytol 205: 149–214. 11336391 10.1016/s0074-7696(01)05004-5

[4] Lee C, Ferguson M, Chen LB (1989) Construction of the endoplasmic reticulum. J Cell Biol 109: 2045–2055. 2478561 10.1083/jcb.109.5.2045 PMC2115887

[5] Wiest DL, Burkhardt JK, Hester S, Hortsch M, Meyer DI, et al (1990) Membrane biogenesis during B cell differentiation: most endoplasmic reticulum proteins are expressed coordinately. J Cell Biol 110: 1501–1511. 2335560 10.1083/jcb.110.5.1501 PMC2200180

[6] Rajasekaran AK, Morimoto T, Hanzel DK, Rodriguez-Boulan E, Kreibich G (1993) Structural reorganization of the rough endoplasmic reticulum without size expansion accounts for dexamethasone-induced secretory activity in AR42J cells. J Cell Sci 105 (Pt 2): 333–345. 10.1242/jcs.105.2.333 7691838

[7] King JC, Williams TH, Gerall AA (1974) Transformations of hypothalamic arcuate neurons. I. Changes associated with stages of the estrous cycle. Cell Tissue Res 153: 497–515. 4474918 10.1007/BF00231543

[8] Carr I, Carr J (1962) Membranous whorls in the testicular interstitial cell. Anat Rec 144: 143–147. 14018858 10.1002/ar.1091440209

[9] Nickerson PA, Curtis JC (1969) Concentric whorls of rough endoplasmic reticulum in adrenocortical cells of the mongolian gerbil. J Cell Biol 40: 859–862. 5765773 10.1083/jcb.40.3.859 PMC2107648

[10] Dykstra KM, Pokusa JE, Suhan J, Lee TH (2010) Yip1A structures the mammalian endoplasmic reticulum. Mol Biol Cell 21: 1556–1568. 20237155 10.1091/mbc.E09-12-1002 PMC2861614

[11] Heidtman M, Chen CZ, Collins RN, Barlowe C (2005) Yos1p is a novel subunit of the Yip1p-Yif1p complex and is required for transport between the endoplasmic reticulum and the Golgi complex. Mol Biol Cell 16: 1673–1683. 15659647 10.1091/mbc.E04-10-0873 PMC1073651

[12] Heidtman M, Chen CZ, Collins RN, Barlowe C (2003) A role for Yip1p in COPII vesicle biogenesis. J Cell Biol 163: 57–69. 14557247 10.1083/jcb.200306118 PMC2173432

[13] Yoshida Y, Suzuki K, Yamamoto A, Sakai N, Bando M, et al (2008) YIPF5 and YIF1A recycle between the ER and the Golgi apparatus and are involved in the maintenance of the Golgi structure. Exp Cell Res 314: 3427–3443. 18718466 10.1016/j.yexcr.2008.07.023

[14] Barrowman J, Wang W, Zhang Y, Ferro-Novick S (2003) The Yip1p.Yif1p complex is required for the fusion competence of endoplasmic reticulum-derived vesicles. J Biol Chem 278: 19878–19884. 12657649 10.1074/jbc.M302406200

[15] Tang BL, Ong YS, Huang B, Wei S, Wong E, et al (2001) A membrane protein enriched in endoplasmic reticulum exit sites interacts with COPII. J Biol Chem 276: 40008–40017. 11489904 10.1074/jbc.M106189200

[16] Matern H, Yang X, Andrulis E, Sternglanz R, Trepte HH, et al (2000) A novel Golgi membrane protein is part of a GTPase-binding protein complex involved in vesicle targeting. Embo J 19: 4485–4492. 10970842 10.1093/emboj/19.17.4485 PMC302084

[17] Calero M, Whittaker GR, Collins RN (2001) Yop1p, the yeast homolog of the polyposis locus protein 1, interacts with Yip1p and negatively regulates cell growth. J Biol Chem 276: 12100–12112. 11278413 10.1074/jbc.M008439200

[18] Yang X, Matern HT, Gallwitz D (1998) Specific binding to a novel and essential Golgi membrane protein (Yip1p) functionally links the transport GTPases Ypt1p and Ypt31p. Embo J 17: 4954–4963. 9724632 10.1093/emboj/17.17.4954 PMC1170824

[19] Chen CZ, Calero M, DeRegis CJ, Heidtman M, Barlowe C, et al (2004) Genetic analysis of yeast Yip1p function reveals a requirement for Golgi-localized rab proteins and rab-Guanine nucleotide dissociation inhibitor. Genetics 168: 1827–1841. 15611160 10.1534/genetics.104.032888 PMC1448722

[20] Kano F, Yamauchi S, Yoshida Y, Watanabe-Takahashi M, Nishikawa K, et al (2009) Yip1A regulates the COPI-independent retrograde transport from the Golgi complex to the ER. J Cell Sci 122: 2218–2227. 19509059 10.1242/jcs.043414

[21] Snapp EL, Hegde RS, Francolini M, Lombardo F, Colombo S, et al (2003) Formation of stacked ER cisternae by low affinity protein interactions. J Cell Biol 163: 257–269. 14581454 10.1083/jcb.200306020 PMC2173526

[22] Koning AJ, Roberts CJ, Wright RL (1996) Different subcellular localization of Saccharomyces cerevisiae HMG-CoA reductase isozymes at elevated levels corresponds to distinct endoplasmic reticulum membrane proliferations. Mol Biol Cell 7: 769–789. 8744950 10.1091/mbc.7.5.769 PMC275929

[23] Chin DJ, Luskey KL, Anderson RG, Faust JR, Goldstein JL, et al (1982) Appearance of crystalloid endoplasmic reticulum in compactin-resistant Chinese hamster cells with a 500-fold increase in 3-hydroxy-3-methylglutaryl-coenzyme A reductase. Proc Natl Acad Sci U S A 79: 1185–1189. 6951166 10.1073/pnas.79.4.1185 PMC345926

[24] Yamamoto A, Masaki R, Tashiro Y (1996) Formation of crystalloid endoplasmic reticulum in COS cells upon overexpression of microsomal aldehyde dehydrogenase by cDNA transfection. J Cell Sci 109 (Pt 7): 1727–1738. 10.1242/jcs.109.7.1727 8832395

[25] Pedrazzini E, Villa A, Longhi R, Bulbarelli A, Borgese N (2000) Mechanism of residence of cytochrome b(5), a tail-anchored protein, in the endoplasmic reticulum. J Cell Biol 148: 899–914. 10704441 10.1083/jcb.148.5.899 PMC2174551

[26] Takei K, Mignery GA, Mugnaini E, Sudhof TC, De Camilli P (1994) Inositol 1,4,5-trisphosphate receptor causes formation of ER cisternal stacks in transfected fibroblasts and in cerebellar Purkinje cells. Neuron 12: 327–342. 8110462 10.1016/0896-6273(94)90275-5

[27] Amarilio R, Ramachandran S, Sabanay H, Lev S (2005) Differential regulation of endoplasmic reticulum structure through VAP-Nir protein interaction. J Biol Chem 280: 5934–5944. 15545272 10.1074/jbc.M409566200

[28] Storrie B, White J, Rottger S, Stelzer EH, Suganuma T, et al (1998) Recycling of golgi-resident glycosyltransferases through the ER reveals a novel pathway and provides an explanation for nocodazole-induced Golgi scattering. J Cell Biol 143: 1505–1521. 9852147 10.1083/jcb.143.6.1505 PMC2132995

[29] Puthenveedu MA, Linstedt AD (2004) Gene replacement reveals that p115/SNARE interactions are essential for Golgi biogenesis. Proc Natl Acad Sci U S A 101: 1253–1256. 14736916 10.1073/pnas.0306373101 PMC337039

[30] Evan GI, Lewis GK, Ramsay G, Bishop JM (1985) Isolation of monoclonal antibodies specific for human c-myc proto-oncogene product. Mol Cell Biol 5: 3610–3616. 3915782 10.1128/mcb.5.12.3610-3616.1985 PMC369192

[31] Kapetanovich L, Baughman C, Lee TH (2005) Nm23H2 facilitates Coat Protein II assembly and endoplasmic reticulum export in mammalian cells. Mol Biol Cell 16: 835–848. 15591128 10.1091/mbc.E04-09-0785 PMC545915

[32] Kalies KU, Rapoport TA, Hartmann E (1998) The beta subunit of the Sec61 complex facilitates cotranslational protein transport and interacts with the signal peptidase during translocation. J Cell Biol 141: 887–894. 9585408 10.1083/jcb.141.4.887 PMC2132780

[33] Ahluwalia N, Bergeron JJ, Wada I, Degen E, Williams DB (1992) The p88 molecular chaperone is identical to the endoplasmic reticulum membrane protein, calnexin. J Biol Chem 267: 10914–10918. 1350281

[34] Hessa T, Meindl-Beinker NM, Bernsel A, Kim H, Sato Y, et al (2007) Molecular code for transmembrane-helix recognition by the Sec61 translocon. Nature 450: 1026–1030. 18075582 10.1038/nature06387

[35] Voeltz GK, Prinz WA, Shibata Y, Rist JM, Rapoport TA (2006) A class of membrane proteins shaping the tubular endoplasmic reticulum. Cell 124: 573–586. 16469703 10.1016/j.cell.2005.11.047

[36] Lorente-Rodriguez A, Heidtman M, Barlowe C (2009) Multicopy suppressor analysis of thermosensitive YIP1 alleles implicates GOT1 in transport from the ER. J Cell Sci 122: 1540–1550. 19383723 10.1242/jcs.042457 PMC2680100

