# Supplementary figures and images for: Identification of Discrete Sites in Yip1A Necessary for Regulation of Endoplasmic Reticulum Structure
Source: PLoS One. 2013 Jan 14;8(1):e54413. doi: 10.1371/journal.pone.0054413 (PMC3544793; doi:10.1371/journal.pone.0054413)

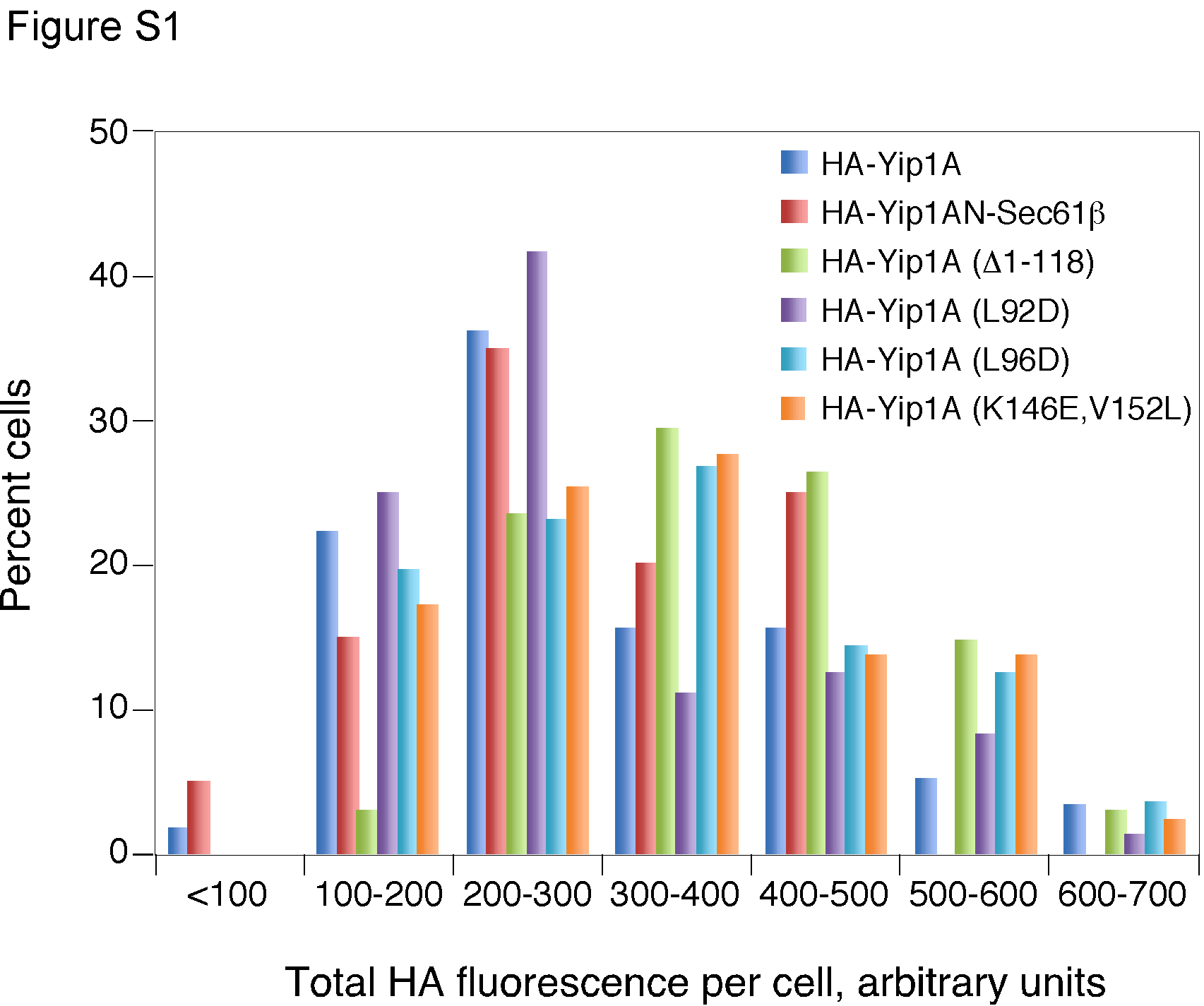

Supplement: Figure S1 — Nonfunctional mutant variants of HA-Yip1A are expressed at levels similar to wild type HA-Yip1A. HeLa cells transfected with the indicated HA-Yip1A variants were fixed 48 h later, stained with antibodies against the HA epitope, and the total fluorescence intensity per cell measured in ImageJ. The data for 50–100 random cells were binned according to levels of fluorescence and plotted in a histogram as the percent of cells exhibiting the indicated levels of fluorescence. (TIF) [file pone.0054413.s001.tif]
